# e-Science and biological pathway semantics

**DOI:** 10.1186/1471-2105-8-S3-S3

**Published:** 2007-05-09

**Authors:** Joanne S Luciano, Robert D Stevens

**Affiliations:** 1 Genetics Department, Harvard Medical School, 77 Avenue Louis Pasteur, Boston, MA 02115, USA; 2 School of Computer Science, Manchester University, Oxford Road, Manchester, M13 9PL, UK

## Abstract

**Background:**

The development of e-Science presents a major set of opportunities and challenges for the future progress of biological and life scientific research. Major new tools are required and corresponding demands are placed on the high-throughput data generated and used in these processes. Nowhere is the demand greater than in the semantic integration of these data. Semantic Web tools and technologies afford the chance to achieve this semantic integration. Since pathway knowledge is central to much of the scientific research today it is a good test-bed for semantic integration. Within the context of biological pathways, the BioPAX initiative, part of a broader movement towards the standardization and integration of life science databases, forms a necessary prerequisite for its successful application of e-Science in health care and life science research. This paper examines whether BioPAX, an effort to overcome the barrier of disparate and heterogeneous pathway data sources, addresses the needs of e-Science.

**Results:**

We demonstrate how BioPAX pathway data can be used to ask and answer some useful biological questions. We find that BioPAX comes close to meeting a broad range of e-Science needs, but certain semantic weaknesses mean that these goals are missed. We make a series of recommendations for re-modeling some aspects of BioPAX to better meet these needs.

**Conclusion:**

Once these semantic weaknesses are addressed, it will be possible to integrate pathway information in a manner that would be useful in e-Science.

## Background

In this paper, we test the semantic integration capability of pathway data for e-Science. In particular, we explore the utility of the BioPAX ontology for providing a *common conceptualization *for the semantics of pathway data and as the mechanism for querying these data. In many aspects, pathway data form the nexus of industrial scale *in silico *biology, and therefore are a microcosm of bioinformatics within e-Science. We use the BioPAX initiative as an exemplar of the kind of semantic activity that enables e-Science to happen and we explore BioPAX's ability to deliver on the requirements of e-Science via use of its BioPAX ontology. The term e-Science is used to describe both the pursuit of global, collaborative *in silico *science and the computational infra-structure to support it. There are several factors present in the development of e-Science. Typically, such endeavors are computationally intensive and are carried out in highly distributed network environments that tend to use large, heterogeneous data sets [1,2]. The infrastructure that supports such science is often referred to as the Grid and promotes virtual laboratories that allow scientists to collaborate and share resources without requiring the location of the scientists or the resources to be in physical proximity. e-Science is intimately linked to Grid computing and bioinformatics is one example of the kind of science that e-Science encompasses [3].

It is easy to see how bioinformatics fits into this paradigm; it has highly distributed and heterogeneous data, along with communities of people working together on biological problems [3]. Indeed, bioinformatics is moving into a more computationally intensive era, with high-throughput experiments being coupled with large-scale systems biology simulations. The scale, in size and complexity, of bioinformatics *in silico *experiments necessitates a transition from hand-crafted single sequence analyses towards systems level approaches. e-Science is bringing about the industrialization of bioinformatics by providing the infrastructure to support these kinds of *in silico *experiments. (Here we take a broad interpretation of *bioinformatics *to include the storage, management and analysis of biological data needed to answer biological questions.)

In 2002, Stein [4] called for the formation of a "bioinformatics nation" through the adoption of technology such as Web services. e-Science exploits these programmatic interfaces to data and uses access to distributed computational resources, often using workflow technology [5-10] to automate the systematic operation of large scale analytical processes.

This kind of industrialization, a result of the high-throughput processing of data and the supporting infrastructure, has already been seen, in particular in the 'omics. The catalogues of the genes present in an organism that are now available for many species in this *post genomic era *has provided the foundation for modeling the interactions of genes and their products in the whole cell. In addition, transcriptome analysis through microarray experiments has produced large volumes of data which have been used effectively and have transformed the field of experimental biology. Coupled with the catalogues of genes, the further layers of 'omic data has begun to provide enough information for the *systems *approach to molecular biology.

### Pathways and systems biology

The factors described above, computationally intensive research, highly distributed network environments, catalogues of organisms' genes, heterogeneous data sets, etc., have come together to create a situation where information about biological networks, of which pathways are fragmentary views, is both the goal of biology and the means through which biologists ask questions.

For most biologists, these networks are conceptualized as fragments or views of parts of the *in vivo *cellular network. These fragments are the commonplace *pathways *seen in numerous papers, presentations and text-books. It is important to remember, however, that they are a conceptualization; simply a way that biologists describe the cellular molecular domain. Nevertheless, they form a point at which genes, gene products, small molecules, reactions, etc. all come together in a *virtual organization*. Pathways are the fundamental form that knowledge must take for the needs of systems biology; the entities in pathways and the data, such as equilibria, rate constants, etc. are all vital for the pursuit of systems biology.

The move towards systems biology has begun to capitalize on the availability of genomic and other information to start mathematically modeling genetic, metabolic and macro-molecular (both regulatory, signaling and metabolic) networks. Data on these networks are recorded as pathways. These pathway models emphasize systems of components rather than the components themselves. It is possible only because the information that has been gathered by molecular biologists over the past twenty years is now readily available.

Pathways are the nexus, the semantic communication point in this endeavor. If we are to model genetic, metabolic and macromolecular networks, then we have to assemble our knowledge from pathway data to reform these networks. e-Science infra-structure is necessary for an industrial approach to systems biology, but it is not in itself sufficient. Unless the data can be co-ordinated, especially at a semantic level, the wealth of biological data will not offer up its knowledge to biologists. It is vital, therefore, that we can reliably interpret the semantics of molecular biological data, particularly at the pathway level.

Unfortunately, pathway information is scattered across many disparate database resources and tends to be conceptualized in different ways, each with its own semantic and syntactic representation. Metabolic pathways capture the precise way in which one molecule is converted into another in a series of biochemical reactions; molecular interaction databases are typically binary relationships, such as protein-protein interactions, or protein-DNA interactions; gene regulatory networks will represent the connections between transcription factors and the genes whose transcription they activate or repress. Signaling pathway representations, which capture the ways in which cells respond to their environment can range from vague or general representations of the form "there's an activation chain in which A activates B activates C" to specific and detailed representations involving a series of complex binding reactions and protein post-translational modifications.

For all these pathway data to be of use to systems biology efforts and e-Science, the following requirements must be met:

• Common conceptual framework. A common model of the entities and their relationships is needed so that all the elements of pathways (pathway steps, reactions, catalysis, large and small molecules, etc. etc.) are all interpreted in the same way.

• Common instances. Once a common conceptualization of the things within the domain of pathways and the relationships among these things is made, the model must be populated. Each resource will have its own conceptual framework that holds data. The same data instances will appear in different resources. When united in one resource it is necessary to know that instances are not redundantly represented in any manner. In database terms this refers to *referential integrity*.

• Common vocabulary. Both the conceptual framework and the instances it holds need a common vocabulary with which to refer to 'things' in the model. This will include common terms for pathways, small and large molecules; atoms and ions; etc.

• Common format. All the data must have the same format. All resources must present the data model and the values within that model in a common form with a common interpretation. XML, RDF, OWL and CSV are all different formats which to a greater or lesser degree can represent data. This refers to the syntax of the language.

If all pathway resources had these aspects in common, it would mean that data from any pathway resource, be it metabolic, regulatory or signaling, could be integrated and interoperated in order to pose research questions.

A large proportion of the concepts that make up the broad definition of the systems biology approach are outlined above. There is, however, no one resource for the network of macro- and small-molecules, together with genes. The numerous databases holding subsets of these data need to be integrated at each level in order to enable systems biology in an e-Science context. In the next section, we describe the BioPAX initiative which aims to define a conceptual framework, or semantic model, that spans these conceptual domain boundaries and includes the definition of the requisite common terms and common data format. This common language would facilitate the exchange and aggregation of pathway knowledge initially, and the assembly of this knowledge in new ways in order to increase the body of scientific knowledge ultimately.

### BioPAX

The BioPAX initiative was undertaken to address the issues of interoperation between pathway data resources. A BioPAX Workgroup oversees the creation of a formal, open-source standard for the representation of biological pathways in a form that can support all pathway data; thus to provide a common conceptual framework, a set of common terms and a common format for exchange and integration. This means that access to all pathway databases in BioPAX format can be achieved with a single parser. Alternatively parsers would be needed by each user for each pathway database. The BioPAX initiative also sought to provide a representation for new data providers and thereby eliminate the mapping of concepts and terms from a native database format to BioPAX.

In support of future uses of pathway data, the BioPAX Workgroup included machine computability among its design principles [11]. This resulted in the choice of OWL-DL. OWL-DL enables full use of reasoners [12], which are software programs that perform inferences based on Description Logics (DL), a subset of first order logic [13]. Furthermore, OWL-DL enables sound and complete inferencing when used by these software reasoners [14,15]. Reasoners can read an OWL file and based on the logical axioms of the OWL ontology, decide whether that set of axioms are logically consistent and, in addition, infer subsumption relationships that are not explicitly encoded in the ontology, for example, it can infer that a certain molecule is a protein. This is a significant advantage over the other representations in the management of knowledge [16].

As OWL was soon to become a W3C recommendation for the standard web ontology language [17], OWL satisfied another BioPAX design goal, namely compatibility. BioPAX, wherever possible, would employ existing standards. In addition, and of great concern to the BioPAX Workgroup was the expressivity of the language. That is, its capacity to represent complex relationships such as those found in biology. For example, in XML-Schema [18] or RDF [19] it is not possible to express that two classes are disjoint, i.e. that a molecule of DNA cannot be a molecule of RNA. It is not possible to express that two classes cannot contain any members in common; that instances that are of the class DNA are not and cannot be of the class RNA. On the contrary, OWL has, for a Description Logic, a wide range of expressivity for describing constraints on class membership by instances [13,16].

To understand the needs of pathway research within e-Science and the issues that arise in meeting these requirements, we present an example at each level of pathway integration mentioned above: conceptual framework, instances, vocabulary, and syntax. First we show how pathway database sources differ at each level, then illustrate how BioPAX addresses these requirements at each level.

#### Different pathway conceptualizations

To illustrate the semantic difference in database conceptualizations, consider the insulin signaling pathway and the glycolysis pathway shown in figures, 1 and 2, from BioCarta [20]. Interactions in signaling pathways are described in terms of a cascade of interacting molecules (or molecular complexes) resulting in a change in some cellular process in response to some stimuli. Each step in the pathway involves a different molecule or molecular complex. Signaling pathways respond to environmental stimuli, either internal or external to the cell, and carry a message that causes (signals) a change in the cell's functioning. Contrast this with a metabolic pathway such as the glycolysis pathway, where one chemical, through a series of precise steps, is transformed into another chemical. In the glycolysis pathway, glucose is transformed into pyruvate. In metabolic pathways, the end product is a transformed chemical molecule, in signaling pathways it is the activation or inhibition of a process.

**Figure 3:**
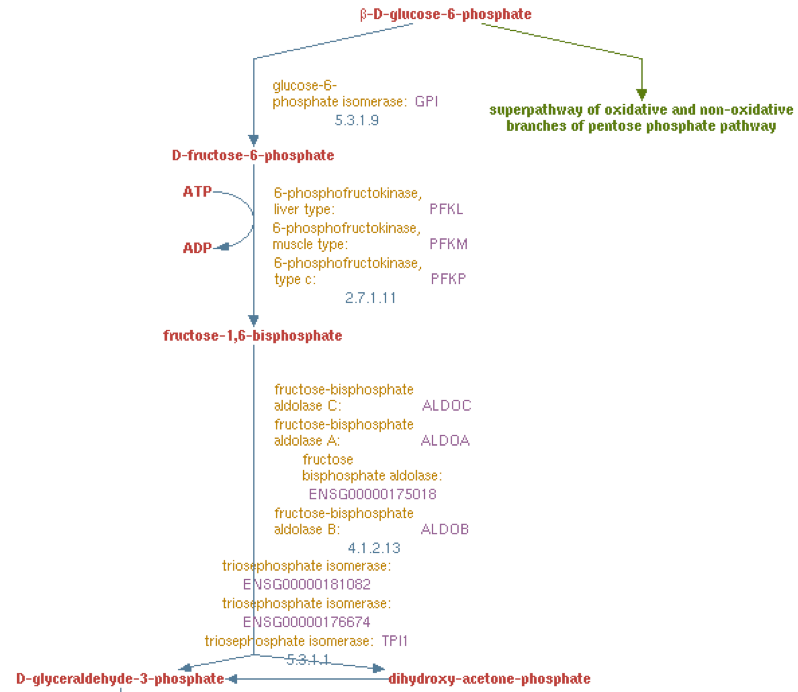
Image showing part of the human glycolysis pathway from HumanCyc.

**Figure 4:**
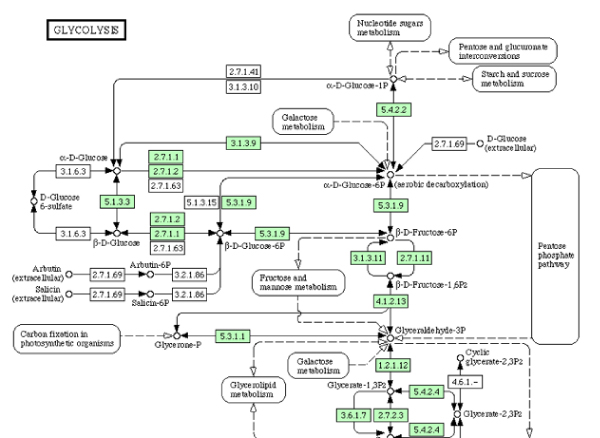
Image showing part of the human glycolysis pathway from KEGG.

While the difference at a conceptual level may be expected between such different categories of pathway types, there are also differences between conceptualizations of pathways of the same type, but defined by different researchers. Because BioCarta does not make a machine readable format available, we will exemplify this with the glycolysis pathway in KEGG [21,22] and HumanCyc [23] (cf. Figures 3 and 4). Just observing the visual representation, it is clear that the pathways start with different molecules and in fact for KEGG there is no obvious starting point. This is one fundamental conceptual difference; the researchers themselves have recognized substantial conceptual differences which have resulted in extensive discussion in the literature [24-27].

To illustrate the issues at the instance, vocabulary, and syntax levels, we focus on the representation of a single reaction, E.C. # 5.3.1.9, and compare its representation in these two databases. One database, HumanCyc, uses the vocabulary term *β*-D-glucose-6-phosphate while the other database, KEGG, uses the vocabulary term *β*-D-Glucose-6P. It is clear that we are referring to the same molecule, i.e. the same real world class of instances, but the vocabulary term used to name these instances differs and while this difference is insignificant for a human reader it is significant for computational processing. The syntax in KEGG is XML and a biochemical reaction is defined as an XML ELEMENT. In KEGG reaction elements have two components, a substrate and a product. The substrate and product elements each have one required attribute, a name which is a KEGG identifier. The data instances in the two resources, for example the representation of *β*-D-glucose-6-phosphate, obviously refer to the same thing. It is, however, hard to do this automatically at the computational level. This semantic integration of data instances (these are not ontologically instances, but represent types or classes of things) is one of the deepest problems in the industrialization of bioinformatics.

In BioCyc, a collection of organism-specific pathway genome databases (PGDB), including HumanCyc, a biochemical reaction is represented as an ENZYMATIC-REACTION in OCELOT [28]. Ocelot is a frame-based system which uses two slots, namely LEFT and RIGHT to represent the reactions participants. The OCELOT syntax is an ASCII flat-file format.

Although there is a clear difference in visual design between the KEGG and BioCyc glycolysis pathway, fundamentally, these are both representations of a biochemical reaction within a metabolic pathway, and as such, the underlying conceptualization of the pathway data they include is very similar, with largely comparable details accessible by clicking through on the interface.

In this section, we have given an example to show what we mean by biochemical reactions being represented differently by different databases at the four levels, conceptualization, instances, vocabulary and syntax. Now we will describe BioPAX followed by an example of how to represent one of the biochemical reactions from HumanCyc in BioPAX. Following this example, you should be able to do the same for a biochemical reaction from KEGG or one of the other databases.

#### Mapping a reaction from HumanCyc to BioPAX

There are two top level classes in the BioPAX ontology: entity and utilityClass. Entities describe the biology while the utility classes are there to record knowledge about the pathway data such as cross references to other databases, evidence codes, and experimental conditions. Pathways are a subclass of entity, along with two sibling classes, interaction and physicalEntity. A pathway has components, PATHWAY-COMPONENTS. The PATHWAY-COMPONENTS will be instances of the class pathwayStep, a utility class. Each pathwayStep contains a set of STEP-INTERACTIONS that describe the physical interactions, such as catalysis, modulation, biochemical reaction, complex assembly, and transport that make up that step in the pathway, or another pathway. A pathway, such as glycolysis, MAPK, or apoptosis is composed of instances of interactions. Interactions can occur between entities so that interactions of interactions and interactions of pathway can be represented. A note on notation: classes in BioPAX use camel-back notation; properties are all upper case with a hyphen separating each word.

**Figure 5:**
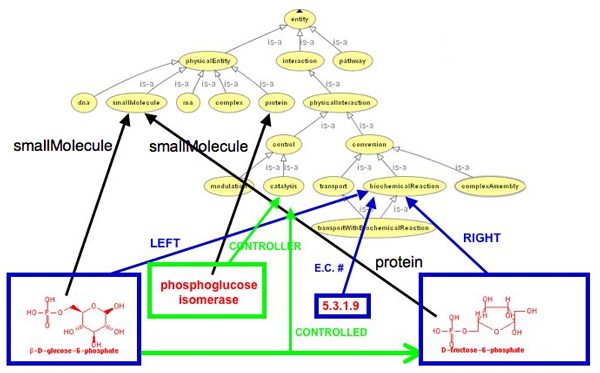
Image showing the ontology and the mapping of a reaction from the glycolysis pathway. The black arrows indicate the physical entity subclasses of the reaction (protein and smallMolecule). The green and blue arrows refer to interaction subclasses. The green arrows indicate the properties of the catalysis class (CONTROLLER and CONTROLLED); the blue arrows indicate the properties of the class biochemicalReaction (LEFT, RIGHT, and E.C.#)

Figure 5 shows how the interactions from one step in the glycolysis pathway are mapped to entity class hierarchy as defined in the BioPAX ontology. A biochemical reaction (across figure at bottom) is mapped to the BioPAX Level 2 root class entity (top of the figure). In this biochemical reaction, there are three instances of the class physicalEntity, of these, two are instances of the class smallMolecule, *β*-D-glucose-6-phosphate and D-fructose-6-phosphate, and one an instance of the class protein, phosphoglucose isomerase, (enzyme is not explicitly represented as a subclass of protein in BioPAX). This biochemical reaction converts *β*-D-glucose-6-phosphate into D-fructose-6-phosphate. The reaction itself is controlled by the enzyme phosphoglucose isomerase. Once the physical entities that participate in the reactions are identified, together with the interaction roles they play in the reaction, we can represent them in BioPAX. Thus in BioPAX, an instance is created of biochemicalReaction with the property LEFT filled with *β*-D-glucose-6-phosphate, the property RIGHT filled by D-fructose-6-phosphate, and E.C.# property filled with 5.3.1.9. An instance of the catalysis class is created with property CONTROLLER filled with phosphoglucose isomerase, and the property CONTROLLED with the reaction name PGLUCISOM-RXN.

#### Pathways in BioPAX

**Figure 6:**
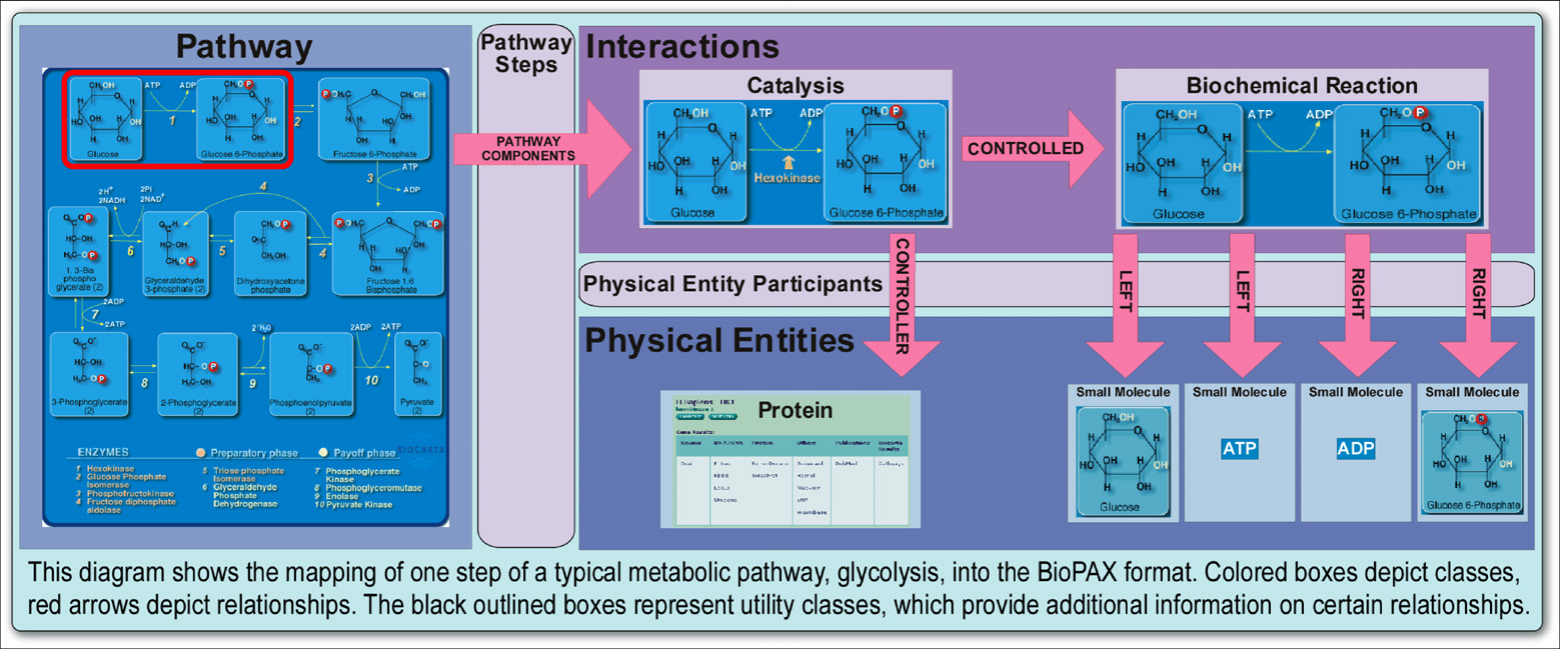
Image showing the mapping of the biochemical reaction *beta-D-glucose*-6-*phosphate *<=>*D-fructose*-6-*phosphate *from the glycolysis pathway in BioCarta to BioPAX [30]. Figure courtesy of Michael Cary and Gary Bader.

In the previous section we describe how a reaction within a pathway is represented in BioPAX. In this section we look at how pathways are represented. In BioPAX, pathways are defined by their components (the property PATHWAY-COMPONENTS). The components of pathways are defined to be any of the classes of interaction, pathwayStep or pathway. Pathway steps are defined as a subclass of utilityClass. The utilityClass is defined in the BioPAX documentation to be a meta-class to assist with the description of pathways, see the discussion section for more on this topic. Each pathwayStep defines a property called NEXT-STEP which provides the order in the pathway and a list of properties that define the set of interactions that occur at that step. This property is named STEP-INTERACTIONS. The interactions at each step can be any subclass of the class physicalInteraction (physicalInteraction was added in BioPAX level 2 as a subclass of interaction in anticipation of level 3 where interactions may also include genetic interactions.) Each of these physical interactions has participants which are instances of the one of the subclasses of physicalInteraction or instances of the class physicalEntityParticipant. The class physicalEntityParticipant is also a utilityClass and is used to describe a physical entity in the context of an interaction. A physicalEntityParticipant specifies the physicalEntity in the context of an interaction by adding the properties CELLULAR-LOCATION and STOICHIOMETRIC-COEFFICIENT. Figure 6 shows the glycolysis pathway from BioCarta [29] with the aforementioned isomerase reaction highlighted and separated into BioPAX classes [30].

#### BioPAX class, instance, vocabulary and syntax

Above we presented the BioPAX class hierarchy and pathway representation. Now we will look at it from the perspective of the levels of integration required for e-Science. First we want to make clear that the conceptualization as described above defines BioPAX as an OWL ontology in terms of its conceptual framework, the instances (called individuals in OWL), the vocabulary terms and the syntax. The syntax used for BioPAX is RDF/XML which is a serialization of OWL. OWL and RDF are graph representations, XML is a tree structure. RDF and OWL must be serialized when written for export. It is important to note that BioPAX defines the classes in which to represent pathway data, the instances are provided by the databases.

The major contribution of BioPAX to e-Science is that it provides a single conceptual framework for the various multiple conceptualizations of pathway databases, i.e. metabolic, molecular interaction, signal transduction and regulatory pathways. It also provides a common format. While the BioPAX ontology has a common terminology at the class level, there is no attempt to provide a common view of instances within those classes. The question then is, now that we have data from different conceptualizations of pathways into the BioPAX representation, can we do in silico science? Can we deliver pathway information to industrial scale analyses? Does BioPAX fulfill the requirements set forth in the introduction?

### An experiment with BioPAX

In this section, we present some experiments using the BioPAX ontology; its instance data and Description Logic reasoning machinery in order to answer questions about pathways. We shall see if BioPAX can match the needs of e-Science outlined in the introduction and the goals of BioPAX itself.

We now have the BioPAX ontology that acts as a common conceptualization for a large quantity of data. These data are encoded as instances of the classes in the BioPAX ontology. In the ontological sense, these are not really instances. For example, we have ATP as an *instance *of the class smallMolecule. The instance in this case and all others in BioPAX data should be classes. We will see later that we have to perform some tricks with BioPAX data because classes are treated as instances.

In OWL, a class represents a set of instances. Classes of instances are described by placing what OWL calls *restrictions *upon classes through combinations of properties and the instances of other classes that act as *successors *to those properties. *Restrictions *are aptly named as they restrict what instances can be members of a class. These restrictions or conditions take two forms:

1. Conditions can be simply *necessary *conditions; that is, any instance must fulfill that condition in order to be a class member. It is not true, however, that fulfilling that condition is enough to recognize an instance as a member of that class. For example, all hexokinase proteins phosphorylate glucose, but the ability to phosphorylate glucose is not enough to recognize an enzyme as a hexokinase.

2. Conditions can be both *necessary *and *sufficient *conditions for class membership; that is not only must any instance of a class hold that condition, but any instance holding that condition or combination of conditions can be recognized by a software reasoner to be a member of the class. For example, the class Enzyme can be defined to be any protein that catalyses a reaction. (This, of course, excludes any catalytic RNA molecules, but that's another story.) Thus, the ability to catalyze a reaction is enough to recognize a protein as an enzyme and all enzymes must catalyze reactions.

As OWL classes describe sets of instances, we can use classes to ask questions of the instance data. Description Logic ontologies have been used in this manner before in systems such as TAMBIS [31]. Here, we wish to ask questions about pathways. Indeed, once we have sets of pathways, we can perform further manipulations upon them to ask further questions. Some example questions a biologist might wish to ask are:

1. What pathways include the vitamin D receptor? That is, what are the pathways that include a step in which the vitamin D receptor participates?

2. What pathways are implied by the gene products of a set of genes? That is, what is the set of pathways that have steps in which a gene product from a set of genes participates?

3. What pathways involve cholesterol? That is, what is the set of pathways in which there is a step that involves cholesterol?

4. What pathways would I expect to see invoked when I add 9-cis-retonic acid to a macrophage? That is, what set of pathways are used in a macrophage and are induced by addition of 9-cis-retonic acid?

5. What pathways exist in a particular cell? That is, what is the set of pathways known to exist in a particular cell type?

6. What pathways exist within particular types of tissues? That is, what is the set of pathways that is the union of all sets of pathways seen in the range of cell types in a tissue?

7. What pathways involve retinoic acid or any of its derivatives (it's oxidative breakdown products)? That is, the set of pathways in which retinoic acid or any of its derivatives is a component of a pathway step?

8. What databases do these instances come from? That is, what is the set of databases from which any of the instances in a set of pathways originate?

9. We have a phenotype, what are the pathways which involve a specified metabolite that might be linked to this effect? That is, what is the set of pathways that is known to be linked to a particular phenotype?

10. What are the metabolites that a set of pathways has in common? That is, what is the set of small molecules that participate in a step of a set of pathways?

11. What is the set of pathways that are active in the particular time interval?

This list is by no means exhaustive, but provides a sample of the kind of questions that one might ask of pathway data. Other researchers who have attempted to ask such questions have used the BioPAX data in its RDF form and used RDF technology, such as RDF stores and RDF query languages to ask these questions [32]. Here we wish to explore the utility of the ontology itself for not only providing some kind of *common conceptualization *for these data but also as the mechanism for querying these data. Current use of the BioPAX ontology does not take advantage of what OWL can do. Rather than a static artifact, we can use the BioPAX ontology as a dynamic, flexible software component. We can use it to recover sets of pathways based on biological descriptions inherent in the BioPAX ontology itself.

## Results

For testing, we used a small subset of the BioCyc BioPAX data, the Argobacterium tumefaciens C58 (ArgoCyc) glycolysis pathway. We chose a subset where we could count the number of instances we expected to retrieve. The following are defined classes, we created, which function as queries over the instance data (OWL individuals):

1. ATPSmallMolecule

2. ATPPhysicalEntityParticipant

3. ATPBiochemicalReaction

4. ATPCatalysis

5. ATPPathwayStep

6. PathwayInvolvingATPBiochemicalReaction

Each of these classes is described such that instances are retrieved based on the involvement of ATP. So, small molecules that are ATP, reactions involving ATP, pathway steps involving ATP and finally pathways involving ATP are all retrieved from the database. The following list shows what was returned for each of the classes defined above:

ATPSmallMolecule

**Returns**:

   smallMolecule127380 ("ATP")

ATPPhysicalEntityParticipant

**Returns**:

   phys-ent-participant127589

            ("6-phosphofructokinase")

ATPPathwayStep

**Returns**:

   pathwayStep127652 ("glycolysis I")

   pathwayStep127583 ("glycolysis I")

   pathwayStep127696 ("glycolysis I")

ATPCatalysis

**Returns**:

   catalysis127540 ("pyruvate kinase")

   catalysis127476 ("phosphoglycerate kinase")

ATPBiochemicalReaction

**Returns**:

   biochemicalReaction127530

            ("Pyruvate kinase")

   biochemicalReaction127468

            ("Phosphoglycerate kinase")

   biochemicalReaction127378

            ("6-phosphofructokinase")

PathwayInvolvingATPBiochemicalReaction

**Returns**:

   pathway127351 ("glycolysis I")

**Note: **The string value of the BioPAX property NAME is shown in parentheses for the readers' convenience. The OWL file used in this example is additional file 1. All files used in this paper needed to be altered from their original version. The modified files are made available on the EMPWR.org website. Please see the section on Debugging the BioPAX Ontology in the Methods section for details about the modifications. Having shown that our approach succeeded with these tests, we wished to query the HumanCyc BioPAX instance data with questions that are more biologically appealing. We developed classes that described the following instances and used data from 2 pathways that were extracted from HumanCyc, the cholesterol biosynthesis pathway and the superpathway of glycolysis, pyruvate dehydrogenase, TCA, and glyoxylate bypass pathway. The files used in this example are additional file 2 and additional file 3.

1. PathwayInvolvingATPBiochemicalReaction or PathwayInvolvingH2OBiochemicalReaction or PathwayInvolvingNADPlusBiochemicalReaction or PathwayInvolvingOxygen2BiochemicalReaction

• pathway79839, pathway17042 (pathway79839 is cholesterol biosynthesis, and pathway17042 is the superpathway of glycolysis, pyruvate dehydrogenase, TCA, and glyoxylate bypass.)

2. CholesterolSmallMolecule

• smallMolecule79898 ("cholesterol")

3. ATPCatalysis

• catalysis17524, catalysis17369, catalysis17161, catalysis17364, catalysis17718, catalysis17511, catalysis17173, catalysis17149, catalysis17353

4. H2OCatalysis

• catalysis17789, catalysis17767, catalysis17772, catalysis17778, catalysis17801

5. NADPlusCatalysis

• catalysis17818, catalysis17328, catalysis17333, catalysis17304, catalysis17279, catalysis17686, catalysis17338, catalysis17289, catalysis17831, catalysis17309, catalysis17274, catalysis17087, catalysis17294, catalysis17299, catalysis17284, catalysis17826

6. Oxygen2Catalysis

• catalysis80292, catalysis80235, catalysis80214, catalysis80101, catalysis80170, catalysis80116, catalysis79970, catalysis80161, catalysis80246, catalysis80152, catalysis80125

The following used the entire HumanCyc data set, which can be found at [33]. As we are working with various builds of Protege4Alpha, performance was an issue and made it impractical to use the entire data set for much of this work.

1. **Class: **PathwayInvolvingCholesterolBiochemicalReaction

• pathway106063

2. **Class: **NoradrenalinePathwayStep

• pathwayStep106053 pathwayStep106054

3. **Class: **PathwayInvolvingNoradrenalineBiochemicalReaction

• pathway106051

4. **Class: **PathwaysInvolvingH2OBiochemicalReaction (HumanCyc contains 185 pathways, of them, 115 involve H2O)

• pathway106411 pathway106531 pathway105873 pathway106934 pathway106008 pathway105463 pathway105937 pathway106460 pathway105742 pathway106804 pathway106785 pathway106682 pathway106296 pathway105696 pathway107053 pathway106919 pathway106379 pathway106331 pathway106791 pathway106477 pathway106150 pathway107067 pathway106123 pathway105810 pathway106321 pathway106451 pathway106689 pathway105689 pathway106851 pathway105642 pathway106544 pathway106002 pathway106421 pathway105825 pathway106354 pathway106057 pathway105681 pathway106037 pathway106829 pathway105734 pathway106394 pathway106206 pathway106249 pathway106565 pathway106306 pathway105667 pathway107061 pathway106585 pathway106365 pathway107134 pathway106717 pathway106656 pathway106581 pathway107152 pathway107079 pathway106553 pathway106384 pathway107093 pathway106814 pathway106389 pathway107017 pathway105704 pathway105715 pathway105798 pathway106571 pathway105955 pathway106284 pathway106268 pathway105792 pathway107031 pathway106645 pathway106776 pathway105457 pathway106337 pathway105452 pathway106908 pathway106807 pathway106833 pathway106981 pathway105885 pathway106195 pathway106416 pathway106432 pathway106499 pathway106428 pathway106729 pathway105473 pathway105727 pathway106596 pathway105677 pathway107180 pathway105721 pathway106467 pathway105655 pathway106548 pathway107036 pathway106219 pathway106939 pathway106399 pathway105979 pathway106756 pathway105966 pathway106494 pathway106347 pathway106441 pathway105674 pathway105928 pathway106671 pathway106664 pathway105971 pathway106028 pathway105684 pathway106015 pathway105757 pathway106634

5. • PathwayInvolvingNADPlusBiochemicalReaction (PathwayInvolvingNADPlusBiochemicalReaction EquivalentTo bp2:pathway and bp2:PATHWAY-COMPONENTS some NADPlusPathwayStep Resutls: 60 pathways)

pathway106389 pathway107017 pathway105798 pathway106531 pathway105873 pathway105955 pathway106460 pathway106750 pathway105742 pathway106268 pathway107053 pathway106645 pathway106776 pathway106919 pathway105457 pathway106379 pathway106130 pathway106150 pathway107067 pathway106765 pathway106451 pathway105810 pathway106689 pathway106981 pathway106902 pathway106954 pathway106170 pathway106421 pathway106416 pathway106432 pathway106428 pathway106729 pathway105825 pathway106037 pathway106596 pathway106394 pathway105734 pathway106467 pathway107172 pathway106219 pathway105667 pathway105979 pathway106585 pathway107134 pathway106365 pathway105966 pathway106486 pathway106441 pathway106656 pathway107079 pathway106970 pathway106949 pathway106553 pathway106617 pathway106671 pathway106028 pathway106384 pathway106015 pathway107093 pathway106634

6. PathwayInvolvingH2OBiochemicalReaction and PathwayInvolvingNADPlusBiochemicalReaction (This is the intersection of the two classes. PathwayInvolvingH20BiochemicalReaction EquivalentTo bp2:pathway **and **bp2:PATHWAY-COMPONENTS some H2OPathwayStep **and **PathwayInvolvingNADPlusBiochemicalReaction EquivalentTo bp2:pathway and bp2:PATHWAY-COMPONENTS some NADPlusPathwayStep)

• Returns the 49 pathways that contain both H2O and NAD+

pathway106389 pathway107017 pathway105798 pathway106531 pathway105873 pathway105955 pathway106460 pathway105742 pathway106268 pathway107053 pathway106645 pathway106776 pathway105457 pathway106919 pathway106379 pathway106150 pathway107067 pathway105810 pathway106451 pathway106689 pathway106981 pathway106421 pathway106432 pathway106416 pathway106428 pathway106729 pathway105825 pathway106037 pathway106596 pathway106394 pathway105734 pathway106467 pathway106219 pathway105667 pathway105979 pathway106585 pathway106365 pathway107134 pathway105966 pathway106441 pathway107079 pathway106656 pathway106553 pathway106671 pathway106028 pathway106015 pathway106384 pathway107093 pathway106634

Our results are correct for the data used. We retrieve all and only those described in the classes. It might seem unusual that only one human pathway involves cholesterol, but that is the only pathway in HumanCyc that appears to involve cholesterol. These results were checked by hand inspecting the RDF data and via the HumanCyc Web site [34]. We also see that once we have sets of pathways, we can quickly derive further sets, for instance by forming the intersection of two sets. This in itself forms a sophisticated way of manipulating pathway data. Ideally, we would like to have used more than one BioPAX compliant resource. Unfortunately, the size of these data running only in memory in the Protégé tool is unsustainable. To combine data from more than one source would mean more work at the start of this process. When defining physicalEntityParticipant, we would have to make more sophisticated definitions of the small molecules and other physical entities that participate in biological processes. We see only one pathway, but HumanCyc contains a series of "reactions without pathways." These reactions map to the following pathways that do appear in KEGG (Table 1). Were we to use both resources at once, we might well retrieve all the appropriate answers.

## Discussion

Our experiments show that we can use the BioPAX ontology to describe sets of pathways. These pathway sets can be further manipulated to derive new sets of pathways. Using the ontology, we can describe these sets according to biological phenomena. This means we can formulate descriptions along the lines of "the set of pathways implied by these genes"; "the set of pathways implied by these up-regulated genes"; etc. This gives a direct link back to the kind of questions described in the section 'An Experiment with BioPAX'. We can answer the majority of the example biological questions outlined in the section 'An Experiment with BioPAX'. The BioPAX ontology has no notion, as yet, of Gene. This means any question with gene as the basis cannot be answered. The development of BioPAX level three should include the notion of gene, so it is likely that this range of question will be able to be formed. The other notable questions that cannot be asked are those involving some temporal aspect. Time intervals are not part of the BioPAX ontology and are absent from much of the data BioPAX attempts to reconcile. The other of our example questions can be answered, even though somewhat clumsily. Any query that is predicated upon using terms from external controlled vocabularies (such as cellular location) are somewhat ugly (in ontological terms). We have not, as yet, explored whether we can answer questions asking for pathways that involve a small molecule or any of its derivatives.

In these experiments we did not actually integrate any data from more than one resource. We only queried data from one resource. Proper integration is our next step. We know this is possible by writing more complex definitions in the current style. This would simply use constructs such as hasName (name-in-db-1 or name-in-db2). This is performing the instance reconciliation we stated as one of the necessary steps for true integration. This approach obviously has its scalability issues. Until such reconciliation is done by the bioinformatics as a whole it will remain as a significant barrier to analytical work.

The points for discussion arising from this work are not so much that it worked, but what we had to do in order for it to work. Examining what we had to do in order to make our approach work, an approach that should be inherent to BioPAX due to its use of a Description Logic, is informative to the future directions of BioPAX.

First, however, a question that should be asked is whether this approach is at all useful or justified? Scalability is an issue. We performed these experiments in memory and this limits the number of instances that can be queried. Tools such as the Instance Store [35] would limit number of individuals to disk space and be, therefore, to all intents limitless. We are aware the current released version of instance Store does not support roles between individuals. Prototype versions do, however, exist that support such constructs. A significant advantage of our approach is the manner of describing the class of instances to be retrieved. Directly modeling the query in the ontology allows the model to be used directly to make the query [31]. The major barrier we encountered in achieving our goal was the ontology itself. As already pointed out [36,37], the BioPAX ontology conflates descriptions of biology and descriptions of the data about biology [32]. It is not that modeling the data is wrong, but doing both at the same time and place in a model makes it difficult to use the description to ask questions. The real issue, however, is exemplified by pathwayStep which is a kind of utilityClass used for purposes of pathway visualization, but also as a real step in a real pathway. If a class is necessary for modeling the data then it should be a utility; if it is needed for describing biology, then it should be in the "biological" part of the ontology. As many of these classes are disjoint (utilityClass and entity, for example) then it becomes very difficult to formulate questions (see section An experiment with BioPAX).

Our definitions are in terms of the data about reality, not the reality itself. We have already observed that defining a class ATPSmallMolecule as an instance that has the name "ATP" is not elegant to say the least (see section 'An Experiment with BioPAX'). ATP is really defined by a certain chemical structure of atoms and covalent bonds. Using the name, however, was one of only two ways we could use the BioPAX instance data to define ATPSmallMolecule. In this experiment, we are restricted to what the BioPAX instance data allowed us to do.

BioPAX allows for use of other controlled vocabularies to label things in its instance data. For example, locations are given by an instance of controlledVocabularyTerm. This sort of works, but what we are stating is that a molecule's location is a term of a controlled vocabulary, not some part of a cell. Much better, in modeling terms, would be to include or refer to external ontologies and use them as ontologies. So, for physical entities, portions of the OBO version of the Chemicals of Biological Interest (ChEBI) dictionary [38] would be introduced. Proteins, RNA and DNA, etc. might have cellular locations provided by the GO's cellular location ontology [39]. In such a case, the actual BioPAX ontology would be very small, but very large by its use of other ontologies. Finally, BioPAX's use of external controlled vocabularies as data items means that the semantic structure of those external vocabularies cannot be exploited. When we ask for pathways involving cholesterol, we retrieve only those involving cholesterol and only cholesterol. This is, of course, fine if that's all we want. In the current situation, asking for cholesterol, kinds of cholesterol or cholesterol derivatives is nigh-on impossible.

What effect would inclusion of external ontology as ontology have? We could, for example, very easily make a class CytosolicKinaseProtein by simply referring, via restrictions, to instances of kinase activity and Cytosol from GO's molecular function and cellular component ontology respectively. Similarly, ATPSMallMolecule, instead of being defined by the name of an individual, would simply be an instance of the ChEBI class for ATP, namely CHEBI:15422. Similarly, we could describe a class of pathways by describing it as performing a biological process of, for instance GO's Glycogen Biosynthesis.

As we describe for the case of defining ATP, we have used what are known as *nominals *(individuals raised to the status of a class) to make class definitions [40]. It would be better modeling practice to put the class ATP as the filler of the definition. In this way we would be saying that, for example, that any instance of a small molecule that has an instance of the structure of ATP is an ATP small molecule. It would be even better to have an ontology of small molecules underneath the BioPAX ontology's smallMolecule class.

Mappings to such an ontology would have to be performed as part of the conversion from client resource to the BioPAX ontology target. Not only would this make the ontological modeling easier, but perform reconciliation at the level of values/instances at the same time.

What are described as "instances" in BioPAX are really classes. All the nominals we used are really classes: ATP; reactions; interactions; pathway steps are really entities that describe sets of instances in the biological world. As described, working at the class level might actually make this work easier. Again, BioPAX has taken a database view of instances. HumanCyc, for example, contains a data instance of ATP. This view has been propagated into the BioPAX ontology and instance data.

We moved pathwayStep to be a kind of pathway. In these experiments this worked. There is an issue, however, of whether a pathway step is a kind of pathway or a part of a pathway. If, in our conceptualization of pathways, we can have a single step pathway, then it is legitimate that a pathwayStep is a kind of pathway. It would also be legitimate (we can find no counter examples) that any one step pathway is part of a greater pathway. In the end, such a choice probably does not make any practical difference.

This brings in some upper level ontology issues. Most upper level ontologies [41-43] make the distinction between Continuant and Occurant (thing and process). Pathways are processes that have physical objects as their participants. Distinctions made at the upper level can sometimes seem a little abstruse, but it helps make basic distinctions, choose one's properties appropriately. It can cause many arguments, but simply sticking to an upper level ontology can aid consistency in modeling decisions.

Based on these discussions and on points raised during this paper, we would advocate the following changes be made to the BioPAX ontology:

1. The use of an upper level ontology to draw distinctions between classes of instances, such as continuants and occurants, independent and dependent continuants etc.

2. That the BioPAX ontology separates descriptions of data from descriptions of biology.

3. The explicit inclusion of other ontologies to provide classes needed to describe the data about pathways.

4. That the instances of BioPAX more reliably represent actual instances rather than classes (see the ATP small molecule example).

5. That a more careful use of OWL's disjoint axiom be used throughout the ontology. The BioPAX ontology has the basis for a well structured ontology based on trees of primitive classes [44]. Along with the other recommendations given here, a combination of pulling apart conflated notions and systematic use of disjunction would give a solid foundation for querying BioPAX data.

6. Careful use of OWL semantics could make explicit some of the semantics held within the comments of the BioPAX ontology.

7. Careful use of OWL semantics, such that there is one BioPAX ontology standard, not two and possibly more with each level release.

## Conclusion

In this paper we have explored the potential for the BioPAX initiative and its ontology to deliver the pathway data necessary for systems style bioinformatics enabled by e-Science. In particular we have looked at the semantics of such integration and how OWL-DL can be used to represent those semantics and be exploited to ask semantically rich questions. We have used the BioPAX ontology to ask questions that deliver sets of pathways. These pathways themselves can then be manipulated to deliver new sets of pathways. The ontology, using its biological aspects, allows sets of pathways or their various components to be described biologically. These sets of pathways can be used as parts of approaches to many analyses. Furthermore, until the BioPAX ontology includes the notions of gene, sequence, chromosome and location, it will not be possible to describe pathways in some of the important ways needed for biological investigations.

The BioPAX initiative succeeds in its basic goal of allowing pathway interoperation. There are now multiple pathway databases, increasing in number, whose data are compliant with the BioPAX ontology. As we described, pathways form a data nexus for modern bioinformatics in an e-Science setting, so this is a significant achievement. The BioPAX ontology acts as a model that allows a wide variety of pathway data to be assembled according to one *common conceptualization *of the domain and in one *common syntax*. It does, however, fail at the level of reconciling pathway *instances*. BioPAX almost provides for this by its inclusion of external controlled vocabularies, but not in the way really desired by this approach to data. Of course, BioPAX does not even attempt to reconcile the labels used for all the entities appearing within pathway data. This is a task beyond its means. We have, however, the technical means to achieve this goal, if not the will and financial means. The current reconciliation makes it possible to query across resources, but a deeper reconciliation would make such querying across resources easier and significantly powerful. Another conclusion to be drawn from this work are ontological conclusions about the BioPAX ontology:

1. The BioPAX ontology conflates modeling of data and modeling of biology.

2. By modeling each separately, a cleaner and easier formulation of queries and description of data would be possible.

3. Raising most instance data to the class level would be a more true reflection of the biological world, but have no absolute effect on the queries possible. In fact, much of the technology would be more effective and modeling would be easier if class things were class things.

4. A modular approach to other ontologies would again reflect the biological world and engender easier queries. Rather than modeling other ontologies as data, importing them as ontologies at the class level would enable richer descriptions to be made during queries. This would have no effect on mapping contributing resources into BioPAX. There are, however, issues of maintenance and synchrony of versions.

5. A closer attention to OWL semantics would make possible a greater range of queries. Problems were caused by the misuse or lack of use of features available in the OWL semantics.

6. Publish one BioPAX ontology; currently there are two separate BioPAX namespaces, one for BioPAX level one and another for BioPAX level 2.

We would advocate, therefore, a re-modeling of the BioPAX ontology. This has to, of course, take the current data providers along. Interestingly, however, much of the re-modeling can be accomplished with little disruption. Many of the current classes are the ones needed. The changes made in these experiments did not necessitate any changes to the data. Simple re-arrangements of the classes are simply reflected automatically in the instance data. New asserted classes would, however, mean changes in instance data encoding.

The BioPAX initiative is an exemplar of the needs of modern, systems level bioinformatics in an industrial, e-Science form. It is typical of bioinformatics data in being highly distributed and highly heterogeneous at all levels. The BioPAX ontology, despite flaws, makes it possible to deliver sets of pathways, described and queried based on biology to bioinformatics analyses.

## Methods

We have the following resources from which to ask these questions:

• The BioPAX ontology.

• The BioPAX data from HumanCyc [23].

• The Pellet [15] and FaCT++ [14] reasoners.

• Protégé 4Alpha [45].

We use both the BioPAX level one and level two ontologies. Any new classes we make, we have placed in a additional file 4. This pays some respect to the BioPAX observance that no new classes should be made; with a module, all our classes can be removed easily.

We wish to form classes that represented at least some of the types of questions listed above. This does, however, break one of the directives in the BioPAX documentation (p.56,57) [11]: "changing the ontology is not recommended if the instance data are meant to be shared.". We ignore this mandate simply because we wish to use the Description Logic machinery with the ontology in order to answer questions. Having built an ontology, provided a large resource of instances, why not use the computational power of OWL-DL? Putting our queries into a separate module means that in effect we do not change the BioPAX ontology as far as any other users are concerned.

In order to answer the kinds of questions listed above, we form new classes that describe sets of entities involved in pathways in the BioPAX instances files. These are sets of pathway instances described in terms of the classes and instances that already exist in the ontology and the instance data. This is typical *conceptual lego *[46] in that the building blocks of the classes already exist and these are built up into more complex class descriptions to answer questions.

The general plan is, therefore, to create some classes that describe pathways. These pathways will be described in terms of, for instance: A catalysis they perform; a macromolecule they involve; a small molecule they involve; etc., as seen in the section BioPAX. The BioPAX ontology describes these types of *entities*.

The BioPAX ontology also has a broad collection of properties or relationships that link the instances of these classes together. In the BioPAX ontology, these have been encoded in two ways:

1. As restrictions on classes. This means that if any instance of the property exists on an instance of the class, then each and every instance of that class must hold that property.

2. As domain constraints. This means that if any instance is found to hold that property, then it is implied to be an instance of the domain class.

This gives a flexible approach to encoding knowledge. The first statement is very strong and the second somewhat weaker, but with potentially profound effects [46]. For example, the BioPAX ontology states that each and every pathway *must *have a NAME. This is in the form of a restriction. The property PATHWAY-COMPONENTS is encoded with domain and range constraints. The domain is pathway and the range is interaction or pathwayStep or pathway. In OWL, this means that any instance that holds a property PATHWAY-COMPONENTS is reasoned to be a member of the class pathway. Note that this is not saying that all pathways must have a pathwayStep or an interaction. In reality they might well do so, but in BioPAX data it is possible for a pathway to be described without there being any data about its component steps or interactions. This is a compromise between modeling some perceived biological reality and modeling the data. Essentially, we can interpret this as meaning "we do not know what the parts of a pathway are, but we know the pathway exists.".

### Encoding BioPAX pathways in classes

Our goal is to describe pathways. The following sequence of connections exist:

• Pathways have pathway components that are pathway steps.

• Pathway steps are made of step interactions.

• Step interactions are made up of interactions (physical interactions such as catalysis, modulation, biochemical reaction, complex assembly and transport, or a pathway).

• Interactions have participants.

• Participants are physical entities of the class small molecule, protein, complex, RNA and DNA.

These classes have other elements to their descriptions, but here we are principally concerned in how to *join together *descriptions to describe sets of pathway instances. We wish to retrieve data that are represented as instances. So, our classes have to have enough description by which to recognize instances as members of the class. This means we have to use conditions that are both necessary and sufficient. We will discuss the ontological soundness of this in the discussion section.

If we wish to have a class for the small molecule ATP, we do the following:

**Class: **ATP

**EquivalentTo: **physicalEntityParticipant

   **that **NAME **value **"ATP"

We are saying that any individual that is a smallMolecule and has the NAME "ATP" is a member of the class ATP. Obviously this is no real definition of the class ATP. It is, however, enough to satisfy the query "give me ATP chemical" in this particular data set. The BioPAX model uses references to the contents of the originating database that detail the primary identifier within a resource for a particular entity. We could use a combination of database identifier and instances identifier to define ATPSmallMolecule (or any other such instance). This could arguably be better modeling, but in this case has the same effect. We can extend our definition to accommodate other data descriptions. All other restrictions on the physicalEntityParticipant class can remain unspecified; they are inherited, but do not need to be specialized unnecessarily. One of OWL's strong points is its ability to model incomplete knowledge [16]. We can use terms from controlled vocabularies to help us define classes of physical entity. We can, for example, describe a class of all physicalEntityParticipant that occur in the cytosol of cells. One of the restrictions on physicalEntityParticipant is that it has a location and that the location is an instance of controlledVocabularyTerm. The BioPAX data has an instance of openControlledVocabulary that is cytosol. By making this restriction both necessary and sufficient, we can make a class with enough description in order to recognize any BioPAX instance that has a cytosolic location. Again, this is not ontologically satisfactory (see Discussion section).

So, we can create physicalEntityParticipant of various kinds. Once we have these classes, we can begin to make more pathway orientated classes. A biochemicalReaction has PARTICIPANTS that are physicalEntityParticipant. The PARTICIPANTS property of biochemicalReaction has two sub-properties, LEFT and RIGHT that describe physicalEntityParticipant on either side of a reaction. Using the super-property, it is easy to describe reactions that involve any kind of physicalEntityParticipant on either side of the reaction in any cellular location. In the example below, bp2: is a prefix used for QNames, the qualified name of the class, which is its URI.

**Class: **ATPSmallMolecule

EquivalentTo:

   bp2:smallMolecule **that**

   bp2:NAME **value **"ATP" string

**Class: **ATPBiochemicalReaction

EquivalentTo:

   bp2:biochemicalReaction **that**

   bp2:LEFT **some**

   ATPPhysicalEntityParticipant

**Class: **ATPCatalysis

EquivalentTo:

   bp2:catalysis **that**

   bp2:CONTROLLED **some**

   ATPBiochemchemicalReaction

**Class: **ATPPEP

EquivalentTo:

   bp2:physicalEntityParticipant **that**

   bp2:PHYSICAL-ENTITY **some**

   ATPSmallMolecule

**Class: **ATPPathwayStep

EquivalentTo:

   bp2:pathwayStep **that**

   bp2:STEP-INTERACTIONS **some**

   (ATPCatalysis **or **ATPBiochemchemicalReaction)

**Class: **PathwayInvolvingATPBiochemicalReaction

EquivalentTo:

   bp2:pathway **that**

   bp2:PATHWAY-COMPONENTS **some**

   ATPPathwayStep

### Debugging the BioPAX ontology

In the previous section we have described how we make the classes that act as our queries. In this section we describe how we make the BioPAX ontology retrieve the appropriate instances. Each time we make a new class, we use the reasoner to check that the ontology is logically consistent; that the new class is placed where we expect it to be placed; and that expected instances from a test set of instances are recovered by the query. At almost every stage of development, we uncovered defects in the BioPAX ontology. Some of these were inappropriate use of OWL and others are deeper ontological modeling issues. Here we list these issues and note the corresponding alterations we made to the BioPAX ontology in order to enable it to function with the Description Logic reasoners.

• BioPAX has two ontologies; level one and level two. Level two extends level one with some extra classes, such as DNA and physicalInteraction. All the classes from level one also appear in the level two module. In OWL, however, a classes identity is its URI; the display label is just a display label. Unfortunately, the classes from level one that are replicated in level two had a different URI and are therefore interpreted as different classes. If the same URI appears in two different modules, the OWL machinery simply works it all out. In this case, however, we find two classes instead of one for all the classes in level one. This was easily fixed by changing the all the URIs to be level two URIs. While this was easily fixed with a global replace, it would be better for the BioPAX ontology to be one ontology.

• There is a major division in BioPAX between utilityClass and entity (see BioPAX section) and these classes are disjoint. One of the classes we used in the construction of new classes (physicalEntityParticipant) is a kind of utilityClass. As physicalEntityParticipant are defined in terms of physicalEntity, this caused the reasoner to return inconsistency errors. Consequently, the physicalEntityParticipant was moved from utilityClass to become a subclass of physicalEntity.

• A similar inconsistency occurred when pathwayStep was analyzed. We made a pathwayStep a subclass of pathway.

• The disjoint axiom between physicalEntityParticipant and physicalEntity was removed.

• The disjoint axiom from pathway and interaction was removed.

• The class physicalEntity was made disjoint with pathway and interaction.

• Many properties have ranges that are other BioPAX classes. For example, the property PHYSICAL-ENTITY has the domain of physicalEntityParticipant. physicalEntityParticipant itself has a restriction of PHYSICAL-ENTITY max 1 owl:Thing. The OWL 1.1 standard only allows cardinality restraints that say any instance holds *n *copies of this property. Implicitly, the instance that *qualifies *this cardinality is owl:Thing. That is, only one property linking an instance of physicalEntityParticipant to an instance of owl:Thing can be present. In theory, this means any instance, a HoverCraft for example, can act as successor to the PHYSICAL-ENTITY property. That is not what what we want. What we want is qualified cardinality restraints, where we specify what type of instance acts as a successor. In this case, we want an instance of physicalEntity to act as successor. Changing BioPAX from the OWL 1.0 cardinality constraint to the more precise qualified cardinality restrictions (QCR) causes inconsistency errors with some reasoners. FaCT++ [14] can reason over QCR that are now part of the proposed OWL 1.1 specification, but Pellet at the time of writing, does not do so [15]. We wished to use QCR in our version of BioPAX so that we could make appropriate definitions. A ProteinPhysicalEntityParticipant is defined in terms of a restriction PHYSICAL-ENTITY max 1 protein. Simple cardinality restrictions would not allow us to do this step that is vital for our needs.

• In biopax-level1.owl, the property DELTA-G is defined as a DatatypeProperty and in biopax-level2.owl, it is defined as an ObjectProperty. The file biopax-level2.owl was modified to use the DatatypeProperty definition. Without this correction, this mistake causes the merged level 1 and level 2 ontologies to be OWL-Full.

• The following two errors cause the reasoner to complain about redefinitions of XML schema types and thus make the ontology OWL-Full (again):

- A fragment of an orphaned range definition needs to be removed from the biopax level 2 file: <owl:DatatypeProperty rdf:about=""/>.

- All occurrences of the string &xsd; need to be replaced with the string xsd:.

Changes were also needed in the HumanCyc.owl file (which contains the instance data) in order to run these experiments:

• The name of the file was changed from biopax.owl to HumanCyc.owl. This was done exclusively for the convenience of the authors who were working with multiple organism and pathway files. SRI, International Inc. The supplier of the BioCyc family of databases reuses the filename biopax.owl for all BioPAX formatted files. Whether they are one pathway or all the pathways within an organism, all are all named biopax.owl. This is only a problem for the human reader; the important name, i.e. the namespace is defined within the file.

• The RDF data type definitions were missing from DELTA-G, MOLECULAR-WEIGHT and STOICHIOMETRIC-COEFFICIENT. This was corrected by adding rdf:datatype=xsd:double to each instance.

• All occurrences of the string level1 were replaced with level2, being the more recent and complete BioPAX ontology. This was an easy work around the problem that BioPAX level 1 and BioPAX level 2 are two independent ontologies (namespaces) notwithstanding the intent to be backward compatible. While this is a BioPAX mistake, it was easier in this case to alter the reference in the data.

Not all of these changes are ontologically valid. Most of them were made simply to get the queries to work. Also, to make the ontology more explicit, we used the QCR feature from the proposed OWL 1.1 extension [47]. Obviously, BioPAX as a project will not use non-standard expressivity. We simply use it here as part of our experiment.

## Competing interests

The authors declare that they have no competing interests.

## Authors' contributions

The authors collaborated in all aspects of the work reported on in this paper.

## Supplementary Material

Additional File 1 ArgoCyc Glycolysis Pathway. This file contains the glycolysis pathway of the organism Argobacterium tumefaciens C58. The file is a subset of the organism specific ArgoCyc database of metabolic pathways from SRI, International. The file was modified from the original as described in the text. Click here for file

Additional File 2 Cholesterol Biosynthesis Pathway in Human. This file contains the cholesterol biosynthesis pathway in humans. The file is a subset of HumanCyc from SRI, International. The file was modified from the original as described in the text. Click here for file

Additional file 3 superpathway of glycolosis, pyruvate dehydrogenase, TCA, and glyoxylate bypass. This tile contains the superpathway of glycolosis, pyruvate dehydrogenase, TCA, and glyoxylate bypass in humans. The file is a subset of HumanCyc from SRI, International. The file was modified from the original as described in the text. Click here for file

Additional File 4 EMPWR BMC Experiment Ontology. This file demonstrates how one can extend the BioPAX ontology by adding additional classes and properties that enable the user to query the pathway instance data. The classes build upon a modified BioPAX level 2. The modified BioPAX level 2 file it builds upon is biopax-level2-EMPWR fresh copy.owl Click here for file

## Figures and Tables

**Figure 1:**
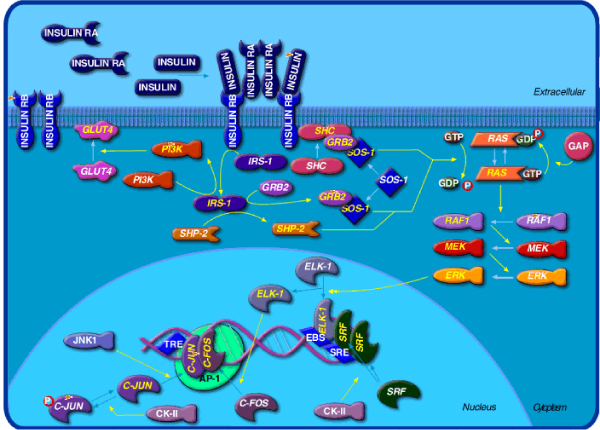
Image showing the human insulin signaling pathway from BioCarta.

**Figure 2:**
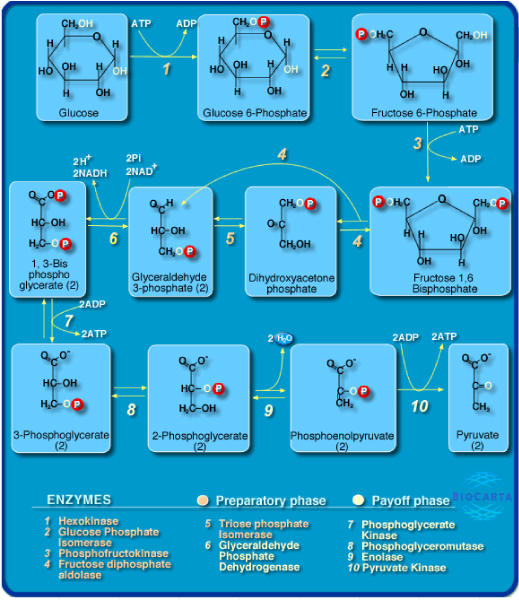
Image showing the human glycolysis pathway from BioCarta.

**Table 1:** The reactions not associated with pathways in HumanCyc but for which there was a corresponding pathway in KEGG.

HumanCyc Reaction	KEGG Pathway and corresponding reaction
cholesterol + an acyl-CoA = cholesterol ester + coenzyme A	R0146 EC 2.3.1.26 in Bile acid biosynthesis pathway
O2 + NADPH + cholesterol = H2O + NADP^± ^+ 7*α*-hydroxycholesterol	R01453 EC 1.14.13.- in biosynthesis of steroids
NADP+ + cholesterol = 7-dehydro-cholesterol + NADPH	R01456 EC 1.3.1.21 in biosynthesis of steroids pathway
O2 + a reduced adrenal ferredoxin + cholesterol = H2O + an oxidized adrenal ferredoxin + 4-methylpentanal + pregnenolone	R02724 EC 1.14.15.6 in C21-Steroid hormone metabolism

The numbers prefixed by R are the reaction numbers in the KEGG database.
